# Evaluating the Clinical Utility of Genome Sequencing for Cytogenetically Balanced Chromosomal Abnormalities in Prenatal Diagnosis

**DOI:** 10.3389/fgene.2020.620162

**Published:** 2021-01-27

**Authors:** Mullin Ho Chung Yu, Jeffrey Fong Ting Chau, Sandy Leung Kuen Au, Hei Man Lo, Kit San Yeung, Jasmine Lee Fong Fung, Christopher Chun Yu Mak, Claudia Ching Yan Chung, Kelvin Yuen Kwong Chan, Brian Hon Yin Chung, Anita Sik Yau Kan

**Affiliations:** ^1^Department of Paediatrics and Adolescent Medicine, Li Ka Shing Faculty of Medicine, The University of Hong Kong, Hong Kong, China; ^2^Department of Obstetrics and Gynaecology, The University of Hong Kong, Hong Kong, China; ^3^Department of Obstetrics and Gynaecology, Queen Mary Hospital, Hong Kong, China; ^4^Prenatal Diagnostic Laboratory, Department of Obstetrics and Gynaecology, Tsan Yuk Hospital, Hong Kong, China

**Keywords:** balanced chromosomal abnormalities, prenatal diagnosis, genome sequencing, long read sequencing, karyotype

## Abstract

Balanced chromosomal abnormalities (BCAs) are changes in the localization or orientation of a chromosomal segment without visible gain or loss of genetic material. BCAs occur at a frequency of 1 in 500 newborns and are associated with an increased risk of multiple congenital anomalies and/or neurodevelopmental disorders, especially if it is a *de novo* mutation. In this pilot project, we used short read genome sequencing (GS) to retrospectively re-sequence ten prenatal subjects with *de novo* BCAs and compared the performance of GS with the original karyotyping. GS characterized all BCAs found by conventional karyotyping with the added benefit of precise sub-band delineation. By identifying BCA breakpoints at the nucleotide level using GS, we found disruption of OMIM genes in three cases and identified cryptic gain/loss at the breakpoints in two cases. Of these five cases, four cases reached a definitive genetic diagnosis while the other one case had a BCA interpreted as unknown clinical significance. The additional information gained from GS can change the interpretation of the BCAs and has the potential to improve the genetic counseling and perinatal management by providing a more specific genetic diagnosis. This demonstrates the added clinical utility of using GS for the diagnosis of BCAs.

## Introduction

Balanced chromosomal abnormalities (BCAs) are changes in either localization or orientation of a chromosomal segment without visible gain or loss of chromosomal material. Such abnormalities can be chromosomal rearrangements, such as translocations, inversions, insertions/excisions, or complex chromosomal rearrangements which are variants that involve more than two breakpoints or more than two chromosomes (De Gregori et al., 2007; Redin et al., 2017). BCAs occur at a significant frequency in both healthy and diseased individuals, affecting about 1 in every 500 newborns (0.2%) (Ravel et al., 2006; Blake et al., 2014). These babies have an increased risk of multiple congenital anomalies, autism spectrum disorders (ASD), or intellectual disability (ID) (Marshall et al., 2008; Blake et al., 2014). In 1991, Warburton performed a study on over 370,000 women who underwent prenatal diagnosis by conventional cytogenetics with the presence of a *de novo* BCA. The observed risks for serious congenital anomalies were 6.1% for reciprocal translocation, 3.7% for Robertsonian translocations, and 9.4% for inversions. Therefore, a figure of 3.7–9.4% is generally quoted as the overall risk of developing a congenital anomaly when a *de novo* BCA is identified (Warburton, 1991).

Conventional karyotyping has been in use for prenatal diagnosis of chromosomal disorders since the 1960s. It can detect numerical abnormalities and structural rearrangements (balanced or unbalanced) but it is restricted to a microscopic resolution of 3–10 Mb (Kirchhoff et al., 2001). Genetic counseling for an apparently *de novo* BCA detected by conventional karyotyping is often challenging. Parents are faced with an estimation of 3.7–9.4% risk that their newborn may develop a range of unknown neurodevelopmental problems or birth defects. The parents have to make a decision on the continuation or termination of pregnancy based on this estimated risk without any more details on how the child can be affected at birth or in the long term (Warburton, 1991). A survey studied the factors affecting the decision making process and found that 27% of parents chose to terminate their pregnancy after a prenatal diagnosis of *de novo* BCA (Wallerstein et al., 2006). Factors affecting the decision to terminate the pregnancy include the type of rearrangement, knowledge of the breakpoints, risk for abnormal outcome, fetal ultrasound findings and anxiety levels. Among all of these, anxiety significantly predicts pregnancy management decisions.

Currently, whole-genome chromosomal microarray (CMA) has been shown to be the preferred first line test for prenatal diagnosis and is a cost-effective tool when applied in the local prenatal diagnostic workflow in Hong Kong (Chung et al., 2020). However, CMA cannot detect balanced chromosomal rearrangements or precise breakpoints at nucleotide resolution which would give additional information on the genetic diagnosis. More recently, massively parallel sequencing-based methods, particularly genome sequencing (GS) has emerged as a comprehensive diagnostic tool. GS has the potential to reveal more cryptic changes than lower resolution techniques with more comprehensive coverage of the genome than targeted techniques, such as exome sequencing. GS can be offered via the more readily available short-read sequencing technique, which has been well-tested in clinical labs with optimization of bioinformatic tools. There are also more advanced methodologies to enhance breakpoint coverage, such as long-read sequencing or optical imaging (Hu et al., 2019; Mantere et al., 2020). Early reports of successful attempts on the use of whole genome paired-end sequencing for breakpoint delineation were demonstrated by Chen et al. who mapped the breakpoints within a region of a few hundred base pairs in four patients with BCA confirmed by long-range PCR and Sanger sequencing (Chen et al., 2010). Subsequently, Redin et al. (2017) investigated the genetic changes in 273 patients with BCAs and congenital abnormalities using GS and found that GS could revise 93% of karyotyping results by at least one sub-band. This study also determined that in BCAs, 33.9% resulted in gene disruptions, 5.2% were involved in pathogenic genomic imbalances, and 7.3% disrupted topologically associated domains (TADs) (Redin et al., 2017).

Although there have been attempts to apply GS on fetuses with BCA in the prenatal setting, GS is not commonly used for clinical practice (Ordulu et al., 2016; Dong et al., 2018; Halgren et al., 2018). As GS sequencing cost continues to be reduced, it is foreseeable that GS will become more affordable for clinical use in the near future. In order to judge the feasibility and clinical utility of GS in the evaluation of BCAs, the clinical implications should be examined. The aim of this study is to assess the clinical utility of GS for BCA in the prenatal setting, and to explore the potential implications on the clinical outcome and decision making process during perinatal period.

## Materials and Methods

### Patient Recruitment

In this retrospective study, subjects with *de novo* BCAs detected via conventional karyotyping at 400–550 band level were identified through the internal database of the Prenatal Diagnostic Laboratory, Tsan Yuk Hospital. A total of twenty prenatal cases were identified in the period of 1996–2017, with ten cases having sufficient DNA for GS and were therefore recruited in this study. Ethics approval has been obtained from the Institutional Review Board of the University of Hong Kong/Hospital Authority Hong Kong West Cluster (UW 18-045).

### Genome Sequencing

Genomic DNA was extracted from the thawed cultured cells of the stored chorionic villi or amniotic fluid samples according to standard protocols. The recommendations of the Laboratory Quality Assurance Committee of the American College of Medical Genetics and Genomics were followed on the use of GS for diagnostic purposes (Rehm et al., 2013). Sequencing was performed by the Illumina HiSeq 2500 platform in Macrogen, Inc or Illumina HiSeq 1500 in the Center for PanorOmic Sciences, the University of Hong Kong. An average of 30 × coverage was targeted for this study.

### Bioinformatic Pipeline and Data Analysis

To detect all chromosomal abnormalities, including cryptic genomic imbalances, GS data were analyzed by in-house bioinformatics pipeline customized for structural variants (SV) and copy number variants (CNV) detection. First, the reads were aligned to the reference human genome build hg19 using Burrows-Wheeler Aligner (BWA) (v0.7.15) (Li and Durbin, 2010). Duplicate marking and sorting was performed using Picard tools (v3.4) (https://broadinstitute.github.io/picard/). SVs were identified by using MANTA (v1.6.0) and LUMPY (v0.2.13) (Layer et al., 2014; Chen et al., 2016). Anomalous read-pairs, defined as paired ends reads that map to two different chromosomes with an abnormal insert-size or unexpected strand orientation, were selected for breakpoint analysis. CNVs were detected by CNVnator (v0.4.1) and NxClinical (v5.2 build 10063) (Abyzov et al., 2011). AnnotSV (Version 2.3) and 3D Genome Browser were used for TAD analysis in the annotation for potential TAD regions impacted (http://3dgenome.fsm.northwestern.edu/) (Geoffroy et al., 2018; Wang et al., 2018).

The SV and CNV identified by the bioinformatics pipeline were manually inspected using the integrated genome viewer (IGV) (Robinson et al., 2011). Visualization of CNVs were also performed using NxClinical. In addition, other orthogonal methods were used for validation of GS results; including Sanger sequencing and PacBio sequencing targeting the junction fragments on the BCA breakpoints for the evaluation of SV, and CMA (Perkin Elmer CGX Oligo Arrays) for the evaluation of CNV. For the SV involving the translocation breakpoint, GS result was determined as “precisely detected” if it was within 20 bp of that of Sanger sequencing. Furthermore, we evaluated the genes disrupted at the translocation breakpoints, genes involved in cryptic deletion or duplication (i.e., CNVs not detectable by karyotyping), and alteration of TADs.

The pathogenicity of gene disruptions was classified with reference to the recommendations for interpreting the loss-of-function mutations (Abou Tayoun et al., 2018). The pathogenicity of CNV was classified according to the latest guideline for interpretation of CNV (Riggs et al., 2020). Supplementary Figures 1, 2 show the overall analysis workflow and TAD of this project, respectively.

## Results

### Patient Demographics

A total of ten subjects were recruited for this study. The prenatal diagnosis of ten cases was performed from 12 to 22 gestational weeks. The indication for prenatal diagnosis included: abnormal ultrasonography (USG) findings (*n* = 6), Down syndrome screening with high risk test results (*n* = 2), advanced maternal age (*n* = 1), and previous pregnancy with chromosomal abnormality (*n* = 1) (Table 1). The pregnancy outcome of the ten subjects ranged from termination of pregnancy (*n* = 3), miscarriage (*n* = 1), and live birth (*n* = 6). Among the six live births, four required further follow-up and assessment. All ten prenatal diagnostic cases had standard G-banded karyotype analysis performed at 400–550 band level (Table 1). There were eight reciprocal translocations and two inversions.

**Table 1:** Comparison of the breakpoint detection by G-banded karyotype and GS on ten BCA cases.

**Case**	**Indication for prenatal diagnosis**	**Outcome**	**Karyotype**	**Sub-band localization of BCA**		**Genomic breakpoints (GRCh37)**	**BCA breakpoint disrupting OMIM morbid gene**	**Cryptic deletion**	**Phenotype associated with the OMIM morbid gene**	**Pathogenicity based on GS^#^**
				**G-banded karyotype**	**GS**					
1	Advanced maternal age; Intracytoplasmic sperm injection	Liveborn at 38 weeks. Birthweight 3.32 kg (75 percentile). Head circumference 35 cm (95 percentile). Referred to clinical genetics for follow up.	46,XX,t(18;19)(q12.2;q13.1)dn	18q12.2 19q13.1	18.q12.1 19q13.12	chr18:29,652,147 chr19:36,930,887	*RNF125*	–	Tenorio syndrome	Variant unknown significance
2	2nd trimester Down syndrome screening T21 risk 1 in 71	Liveborn at 40 weeks. Birth weight 3.2 kg. Anal polyp found at birth with ligation done at 3 months. No follow up at pediatrics or genetics.	46,XX,t(4;12)(q35;p13.1)dn	4q35 12p13.1	4q35.2 12p12.3	chr4:186,776,072 chr12:15,513,829	–	–	–	Negative
3	Alpha thalassemia couple; USG showed increased cardio-thoracic ratio	Medical termination of pregnancy at 18 weeks for Hb Bart syndrome	46,X,inv(X)(p21q22.1)dn	Xp21 Xq22.1	Xp21.1 Xq22.1	chrX:34,271,812 chrX:99,594,533	*PCDH19*	–	X-linked early infantile epileptic encephalopathy	Likely pathogenic
4	Previous termination of pregnancy with absence of cerebellar vermis and corpus callosum, *de novo* 1p32.1–1p31.3 deletion.	Liveborn at 39 weeks. Birth weight 3.15 kg. Mild global developmental delay, mild hypotonia, phonological delay. Last developmental assessment at 5 years 10 months: development close to age. Study normal school and remained well at 7 years	46,XY,t(5;9)(q13;q32)dn	5q13 9q32	5q14.1 9q32	chr5:78,899,784 chr9:117,899,085	–	–	–	Negative
5	1st trimester Down syndrome screening T21 risk 1 in 100	Liveborn at 37 weeks. Birthweight 2.78 kg. Last developmental assessment at 5 years 4 months: normal development with borderline social, language and practical reasoning delay, mild disarticulation and ascent, mild ASD features, referred for ASD workup now 6 years old	46,XY,t(1;4)(p21;q21.1)dn	1p21 4q21.1	1p21.2 4q21.1	chr1:100,004,147 chr4:88,309,179	–	–	–	Negative
6	1st trimester Down syndrome screening T21 risk 1 in 18; increased nuchal translucency 5.92 mm	Silent miscarriage with hydropic features at 15 weeks. Medical induction	46,XX,t(8;11)(q22;q13)dn	8q22 11q13	8q22.1 11q13.1	chr8:97,086,794 chr11:65,540,889	–	–	–	Negative
7	1st trimester Down syndrome screening T21 risk 1 in 50; USG showed round head shape	Medical termination of pregnancy at 21 weeks. No postmortem. Chromosome microarray showed arr[GRCh36] 12q15q21.1(68998005_(70431136)x1 dn	46,XY,t(7;18;12)(q31;p11.3;q15)dn	7q31 18p11.3 12q15	7q31.1 18p11.31 12q15	chr7:110,493,772 chr18:3,766,591 chr12:69,794,115	–	1.5 Mb deletion in chromosome 12 involving *CNOT2*	12q15 deletion syndrome	Pathogenic
8	USG showed early-onset intrauterine growth restriction	Livebirth 35 weeks. Birthweight: 1.06 kg MOPD II	46,XY,inv(21)(q11.2q22.3)dn	21q11.2 21q22.3	21q11.2 21q22.3	chr21:14,953,345 chr21:47,839,992	*PCNT*	–	Microcephalic osteodysplastic primordial dwarfism type II (MOPDII)	Pathogenic
9	USG showed intracardiac echogenic focus, short long bones	Liveborn at 40 weeks. Birthweight 3.1 kg. Normal newborn examination. Mild global delay since early infancy with autistic features. Autism spectrum disorder when 6 years old Follow up psychiatry and Clinical genetics.	46,XY,t(6;8)(p21.1;q24.1)dn	6p21.1 8q24.1	6p21.1 8q24.21	chr6:45,841,060 chr8:126,545,471	–	–	–	Negative
10	USG showed left cleft lip	Medical termination of pregnancy at 21 weeks. Postmortem: left cleft lip. Chromosomay microarray showed arr[GRCh37] 7q36.2q36.3(153873199_158608579)x1 dn	46,XX,t(3;7,6)(q25;q36;q21)dn	3q25 6q21 7q36	3q25.3 6q21 7q36.3	chr3:157,254,970 chr6:106,600,067 chr7:158,852,085	–	4.9 Mb deletion in chromosome 7 involving *DPP6, MNX1* and *SHH* gene	Autosomal dominant intellectual disability 33, Currarino syndrome and holoprosencephaly	Pathogenic

# *Classification based on ACMG/AMP or ACMG/ClinGen guideline. USG, ultrasound findings*.

### Diagnostic Performance of GS

All breakpoints of the BCAs identified by karyotyping were detectable by GS. The average coverage of the ten GS was 35.9 × and the percentage of coverage over 20 × was 93.1%. Table 1 shows the comparison between karyotyping and GS in the detection of BCA chromosomal sub-band changes. With the detection of breakpoints at the nucleotide level, GS identified gene disruption in three cases and genomic imbalances surrounding the BCA breakpoints in two cases.

GS refined the breakpoints at the chromosomal sub-band level in all cases except case 8 where no revision of the breakpoints was necessary, as karyotype identified the correct sub-band. Orthogonal methods were used for validation in a total of eight cases. Sanger sequencing (*n* = 5) and PacBio sequencing (*n* = 1) were used for the validation of breakpoints in 6 cases (Supplementary Table 1), and CMA was used as validation of deletions in another two cases (Case 7 and 10). The primers of Sanger sequencing are shown in Supplementary Table 2. Sanger sequencing confirmed all (*n* = 5) BCA breakpoints determined by GS were within 20 bp of the true mutation (Supplementary Figure 3). PacBio sequencing also confirmed the BCA breakpoint within 1 bp of Illumina short read detection (Case 8).

Copy number variation (CNV) surrounding the BCA breakpoints were analyzed and two copy number loss overlapping the BCA breakpoints (Supplementary Table 3) were detected on two samples (Case 7 and 10). Both CNV deletions were concordantly detected by CNVnator and NxClinical as described. No TAD domain disruption was found in this study. Using the ACMG/AMP and ACMG/ClinGen pathogenicity framework, GS achieved a pathogenic molecular diagnosis in four out of ten patients resulting in a diagnostic yield of 40%. There was one variant of unknown significance and five variants that did not disrupt any coding genes (Table 1). Three cases with gene disruption and two cases of genomic imbalances are described in detail with pathogenicity interpretation.

### Gene Disruption

Case 1: A 40-years-old Chinese woman had a history of ectopic pregnancy and bilateral salpingectomy for hydrosalpinx. She underwent *in-vitro* fertilization and intracytoplasmic sperm injection for male and tubal factor subfertility. She then had one first trimester silent miscarriage requiring suction evacuation. For the index pregnancy, amniocentesis was performed at 18 weeks which showed 46,XX,t(18;19)(q12.2;q13.1)dn. She had gestational diabetes with satisfactory control on diet. A female baby of 3.32 kg (75th percentile) was delivered by lower segment Cesarean section at 38 weeks due to breech presentation. The newborn examination was normal. The head circumference was 35 cm (95th percentile). The birth length was not available. The baby was referred to clinical genetics in view of *de novo* reciprocal translocation but was absent for follow up. GS showed the translocation breakpoints were at chr18:29,652,147 and chr19:36,930,887. This was validated by Sanger sequencing (Supplementary Figure 3A). The translocation breakpoint in chromosome 18q disrupted the *RNF125* gene on exon 6. *RNF125* is associated with Tenorio syndrome (OMIM 616260), an autosomal dominant disease characterized by overgrowth, macrocephaly, and intellectual disability. The gene has not been curated by ClinGen yet. *RNF125* is predicted to be tolerant of loss-of-function mutations by a low pLI score of 0. In addition, all reported pathogenic variants in *RNF125* are missense mutations therefore the gene disruption is not compatible with the disease mechanism. According to the ACMG/AMP Variant Interpretation Guidelines, the variant was classified as a variant of uncertain significance.

**Figure 1:**
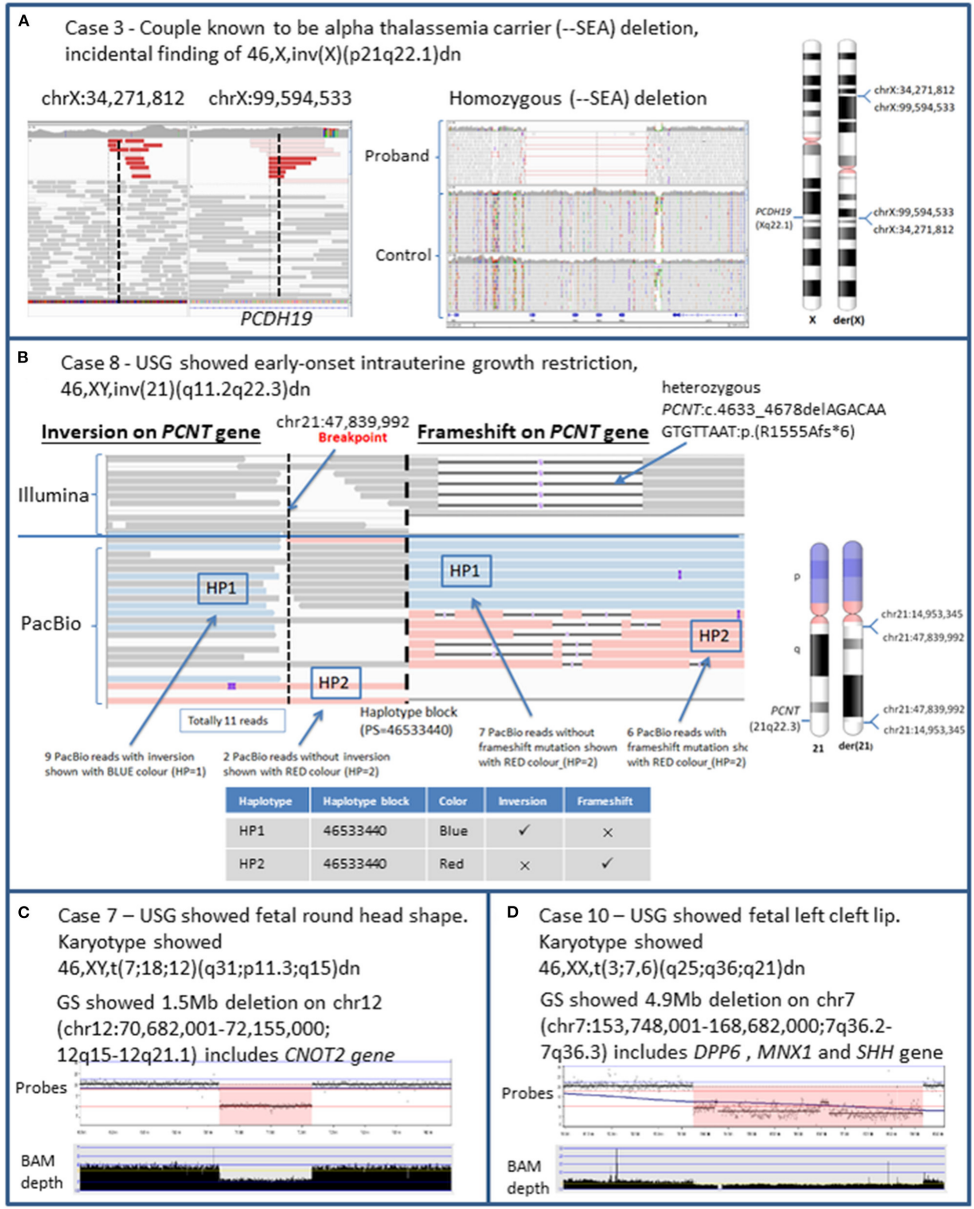
Detection of structural variations and cryptic CNV using genome sequencing in prenatal diagnosis. The above ideograms of G-band pattern of human chromosomes are in 550 bphs (bands per haploid set). **(A)** The fetus was known to be affected with Hb Bart syndrome at 18 weeks by genetic testing for alpha-globin genes (*HBA1, HBA2*). Incidental finding of 46,X,inv(X)(p21q22.1)dn was detected. WGS detected the homozygous (--^SEA^) deletion and the inversion breakpoint that resulted in the gene disruption of *PCDH19* (OMIM 300460) at exon 6. Haploinsufficiency of *PCDH19* is associated with the disease Epileptic encephalopathy, early infantile, 9 (OMIM 300088). **(B)** USG showed early-onset intrauterine growth restriction. Amniocentesis at 22 weeks showed 46,XY,inv(21)(q11.21q22.3)dn. The IGV plot represented regions near the breakpoint (chr21:47,839,992) of the inversion on the *PCNT* gene at intron 31 and heterozygous frameshift mutation on *PCNT*:c.4633_4678delAGACAAGTGTTAAT:p.(R1555Afs*6). The upper track shows the Illumina reads while the bottom track shows the PacBio reads. The Illumina track shows there was an inversion breakpoint and a frameshift mutation on *PCNT* gene but phasing information could not be known due to the short read length of 150 bp available. The PacBio track showed the two haplotypes in blue color (HP1) and red color (HP2) at the same Haplotype block (PS = 46533440) generated by WhatsHap. Combining the Illumina, PacBio, and WhatsHap this showed that the inversion breakpoint (blue color) was in trans with the frameshift mutation (red color) which explained the autosomal recessive inheritance of the genetic condition MODPII. **(C)** USG showed fetal round head shape. Illumina GS data illustrated by NxClinical genome viewer showed there was a 1.5 Mb deletion on chromosome 12 (chr12:70,682,001–72,155,000; 12q15–12q21.1) involving a partial deletion of the 3′ end of *CNOT2* (OMIM 604909) which is associated with 12q15 deletion syndrome (OMIM 618608). The copy number loss was also detected on chromosome 12 via CMA arr[hg18] 12q15q21.1(68998005_70431136)x1. The detection of the genomic imbalance (12q15–12q21.1) by GS was comparable with CMA. **(D)** USG showed fetal left cleft lip. Illumina GS data illustrated by NxClinical genome viewer showed there was a 4.9Mb deletion on chromosome 7 (chr7:153,748,001–168,682,000; 7q36.2–7q36.3) involving a partial deletion of the 3′ end of *DPP6* (OMIM 126141), whole gene deletion of *MNX1* (OMIM 142994), and *SHH* (OMIM 600725) gene was associated with the Autosomal dominant intellectual disability 33 (OMIM 616311), Currarino syndrome (OMIM 176450) and Holoprosencephaly (OMIM 142945), respectively. CMA was performed and detected a copy number loss on chromosome 7 (chr7:153,748,001–158,682,000) arr[GRCh37] 7q36.2q36.3(153873199_158608579)x1. The detection of this copy number loss by GS was comparable with CMA.

Case 3: A 34-years-old Chinese woman and her husband were alpha thalassemia carriers. She had one healthy child and a history of termination of pregnancy due to hemoglobin Bart hydrops fetalis. For the index pregnancy, she underwent amniocentesis at 17 weeks because the ultrasound examination showed mild cardiomegaly with increased cardio-thoracic ratio. Genetic testing for alpha-globin genes (*HBA1, HBA2*) showed the fetus was homozygous for --^SEA^ deletion, confirming the molecular diagnosis of Hb Bart syndrome. Karyotype showed 46,X,inv(X)(p21q22.1)dn. Medical termination of pregnancy was performed. GS identified the --^SEA^ deletion confirming the molecular diagnosis. GS also identified the breakpoints at chrX:34,271,812 and chrX:99,594,533 which resulted in the disruption at exon 6 of *PCDH19* gene. ClinGen curated *PCDH19* gene with sufficient evidence for haploinsufficiency showing loss of function is the disease-causing mechanism. The gene is associated with X-linked early infantile epileptic encephalopathy (OMIM 300088) (Figure 1A) which is characterized by seizure onset in infancy and mild to severe intellectual impairment. It mainly affects heterozygous females whilst carrier males are largely unaffected except for minor psychiatric/behavioral abnormalities (Jamal et al., 2010). Using the ACMG/AMP guideline for variant interpretation, the variant was classified as pathogenic (Table 1). If the baby was born, she would have a high chance of developing early-onset severe epilepsy and encephalopathy given the full penetrance of the condition.

Case 8: The first pregnancy of a 30-years-old Chinese woman was noted to have early onset intrauterine growth restriction (IUGR) since 18 weeks. Congenital infection screening was negative and morphology scan was normal. Amniocentesis at 22 weeks showed 46,XY,inv(21)(q11.21q22.3)dn and normal chromosomal microarray results. Serial ultrasound examination showed severe IUGR and short long bones. The baby was delivered by emergency lower segment Cesarean section due to antepartum hemorrhage of unknown origin at 35 weeks. His birth weight was 1.06 kg (<3rd percentile), body length was 34.5 cm (<3rd percentile), and head circumference was 28 cm (<3rd percentile). Skeletal survey at 1 month showed mesomelic limb shortening. On follow up, microcephaly became more prominent with subtle craniofacial dysmorphism including sloping forehead, small ears, upward slanting palpebral fissures, and prominent nasal root. Other clinical features included global developmental delay, severe subglottic stenosis, and recurrent vomiting. At 2 years of age, assessment by clinical geneticist led to a clinical suspicion of microcephalic osteodysplastic primordial dwarfism type II (MOPDII), an autosomal recessive condition caused by *PCNT* mutations. Exome sequencing was performed and identified a likely pathogenic, maternally inherited frameshift mutation in *PCNT* [c.4633_4678delAGACAAGTGTTAAT p.(R1555Afs^*^6)], yet the second mutation cannot be found. Subsequently, immunofluorescence analysis was performed and pericentrin protein was absent in skin fibroblast, suggesting the presence of biallelic loss-of-function *PCNT* mutations. Given that the inversion occurred on chromosome 21, it was suspected that inversion resulted in the second mutation. GS was performed and revealed that the inversion disrupted intron 31 of *PCNT* at chr21:47,839,992. Since the inversion breakpoint and frameshift mutation were 20,440 bp apart, PacBio long read sequencing was used to determine the phasing of the mutations. Using both short-read and long-read GS with the bioinformatic tool WhatsHap (Supplementary Figure 4), it was determined that the inversion breakpoint and the frameshift mutation were located on different haplotypes, confirming the molecular diagnosis of MOPDII (Figure 1B, Supplementary Figures 5, 6) (Martin et al., 2016). The child attended regular follow up at age of 7 with mild intellectual disability, features of attention deficit hyperactivity disorder, hypogonadism, insulin resistance, and undescended left testes. His lipid profile remained normal.

### Genomic Imbalances

Case 7: A 28-years-old Chinese woman had chorionic villi sampling performed at 13 weeks gestation for high risk first trimester Down syndrome screening test result. Karyotype shows a three-way complex chromosomal rearrangement 46,XY,t(7;18;12)(q31;p11.3;q15)dn. GS identified a 1.47 Mb copy number loss on chromosome 12 ([hg19] chr12:70,682,001–72,155,000, Figure 1C). CMA (Perkin Elmer CGX Oligo Arrays) also detected a 1.43 Mb interstitial deletion on chromosome 12. This deletion is likely related to cryptic imbalances resulting from one of the breakpoints of the three-way translocation. Subsequent anomaly scan showed a round head shape. This copy number loss causes a partial deletion of the 3′ end of the *CNOT2* gene up to intron 3. ClinGen has yet to curate *CNOT2* for haploinsufficiency. *CNOT2* is associated with an autosomal dominant intellectual developmental disorder with nasal speech, dysmorphic facies, and variable skeletal anomalies (OMIM 618608). The gene is predicted to be haploinsufficient with a haploinsufficiency score (HI index) of 4.39 and pLI score of 1. Studies have characterized 12q15 microdeletion syndrome to *CNOT2* as the prime candidate with multiple *de novo* cases causing similar phenotype (Alesi et al., 2019). Using the ACMG/ClinGen pathogenicity recommendation for CNV, the copy number loss was determined to be pathogenic (Supplementary Table 3).

Case 10: A 37-years-old Chinese woman had an anomaly ultrasonography scan showing fetal cleft lip at 21 weeks. Amniocentesis was performed with karyotyping and detected a translocation at 46,XX,t(3;7,6)(q25;q36;q21)dn. GS identified a 4.93 Mb copy number loss on chromosome 7 (chr7:153,748,001–158,682,000, Figure 1D). CMA (Perkin Elmer CGX Oligo Arrays) also detected a 4.74 Mb interstitial deletion on chromosome 7 arr[GRCh37] 7q36.2q36.3(153873199_158608579)x1 which likely resulted from cryptic loss adjacent to one of the BCA breakpoints. This copy number loss causes a partial deletion of the 3′ end of the *DPP6* up to intron 1 and whole gene deletions of the *MNX1* and *SHH* genes. ClinGen curated both *MNX1* and *SHH* genes with sufficient evidence for haploinsufficiency whilst *DPP6* gene only had minimal evidence for haploinsufficiency. The *MNX1* gene is associated with Currarino syndrome (OMIM 176450), an autosomal dominant syndrome characterized by association of partial sacral anomalies, a presacral mass, and anorectal malformations. The *SHH* gene is associated with the autosomal dominant disorder holoprosencephaly 3 (OMIM 142945). *DPP6* is associated with autosomal dominant intellectual disability 33 (OMIM 616311). The whole gene deletion of two haploinsufficient genes allowed for the classification of this copy number loss as pathogenic (Supplementary Table 3) (Crétolle et al., 2008; Solomon et al., 2012). Postmortem results show the fetus had left cleft lip, which may be supportive of holoprosencephaly, however, other facial features were unremarkable, the cerebral sulci and gyri are not yet formed, the anus was patent, and there are no obvious abnormality over the back or sacral area to be suggestive of midline abnormality.

## Discussion

This study has shown that GS enabled accurate identification of all breakpoints in the ten cases with BCA. In nine out of ten cases in this study (90%), the conventional karyotype results were revised by at least one sub-band. This result is comparable with a study by Redin et al. showing at least one sub-band could be revised in 93% of subjects when compared to karyotype (Redin et al., 2017). Out of 10 cases, three BCA cases (Case 1, 3, and 8) had breakpoints causing gene disruptions and two BCA cases (Case 7 and 10) causing genomic material gain/loss at the breakpoints. In these five cases, four (Case 3, 7, 8, and 10) were classified as pathogenic under the ACMG pathogenicity framework. Although the other one BCA (Case 1) caused gene disruption, its clinical significance remained uncertain. After GS of all ten cases, no gene disruption, cryptic deletion, or TAD disruption was detected in the remaining five cases (Case 2, 4, 5, 6, and 9). Validation via orthogonal methods or PacBio sequencing was performed in 8 of 10 cases (Case 1, 2, 5, 6, 7, 8, 9, and 10) where sufficient DNA remained (Supplementary Figure 3). The analysis showed that the accurate determination of BCA breakpoints using GS allowed a better estimation on the pathogenicity of the BCA.

Overall, the information obtained from GS led to improved clinical utility by giving a definitive molecular diagnosis in four out of ten cases (Cases 3, 7, 8, and 10). In case 3, the parents were known carriers of alpha thalassemia and their USG showed an increased cardiothoracic ratio of the fetus. Genetic testing for alpha-globin genes (*HBA1, HBA2*) were performed based on the family history and the prenatal molecular diagnosis of Hb Bart's hydrops fetalis syndrome (BHFS) was made by single gene testing. Genetic counseling was given to the parents regarding how BHFS was once considered a fatal disease but survival is possible, and the possible short and long term complications were discussed (Chan et al., 2018). Although the final decision was medical termination, the continuation of pregnancy with intrauterine transfusion was a possible option, as shown by nine reported cases locally in Hong Kong and 69 cases in the BHSF International Consortium (Songdej et al., 2017; Chan et al., 2018). Nevertheless, we show that if GS was performed initially, further information could be given by a dual diagnosis of homozygous --^SEA^ deletion for Hb Bart syndrome and X-linked early infantile epileptic encephalopathy. While HBFS alone requires cautious counseling, the identification of the X-linked early infantile epileptic encephalopathy with high penetrance will also mean there is the added risk of infantile onset seizures and mild to severe intellectual impairment. This additional information would allow a more comprehensive evaluation of the possible long term outcomes of the child's development and a more informed discussion regarding the pregnancy decision could be made.

In another complex situation, case 8, the diagnostic journey was long and laborious. The fetus originally presented with IUGR, a common antenatal finding. Molecular tests offered prenatally included conventional karyotyping and CMA. Postnatally, exome sequencing was offered but a molecular diagnosis was not reached. Short read GS was further offered as part of this project. The use of GS facilitated the diagnosis but required another complementary sequencing technology (PacBio sequencing) to fully characterize the underlying genetic defect. The confirmation of MOPDII required a series of postnatal clinical assessment and several genetic tests. The information from the combination of short read and long read GS was essential for the genetic counseling of case 8. The molecular diagnosis of MOPDII with autosomal recessive inheritance was determined by a maternally inherited allele and a second *de novo* BCA confirmed by long read GS. The *de novo* mutation gives important information for counseling as the risk of recurrence in the next pregnancy is reduced. This additional sequencing work to identify the second *de novo* mutation was worthwhile because it minimizes distress for the family regarding the source of the second mutation and facilitates better counseling and family planning.

Detection of genomic imbalances surrounding BCA breakpoints through GS identified two pathogenic variants in this study. In both cases, the *de novo* BCAs are complex chromosomal rearrangements (Madan et al., 1997). In both cases, the use of GS allowed more accurate breakpoint delineation from whole gene deletion to partial gene deletion. Although in these two cases the conclusion of a loss of function effect of the gene did not change the clinical interpretation, the more accurate breakpoint delineation may imply significant differences in clinical interpretation. For example, accurate detection could translate into completely different genetic syndromes, e.g., Marshall-Smith syndrome or overgrowth syndrome for different exons of the *NFIX* gene (Martinez et al., 2015). In some cases, copy imbalances at the 3′ end can be benign, such as duplications of the *DPP10* gene in autism spectrum disorders (Mak et al., 2017). Such genomic imbalances are important in BCAs with multiple translocations, and such pathogenic changes have previously been described in complex chromosomal rearrangements (Madan et al., 1997; De Gregori et al., 2007). While CMA is a well-established tool in the detection of copy number changes, the higher resolution provided by GS will provide additional insights into the precise impact on gene function and would be able to detect deletions not covered by the resolution of CMA.

A negative GS result can also benefit genetic counseling as a reduced risk of congenital anomalies can potentially be determined. An absence of OMIM genes at the BCA breakpoints can give additional reassurance to the patients beyond the standard 3.7–9.4% risk determined by karyotyping. When used routinely, this information can help reduce anxiety of the family when interpreting BCA findings.

With the accurate detection of BCA breakpoints using GS, pathogenic variants were identified in four of the ten cases. The detection rate of higher resolution genomic changes was improved to 40%. A systematic review of literature for short-read GS used on prenatal samples with a similar inclusion criteria to our study has yield similar results (Supplementary Figure 7). The overall morbidity of congenital anomalies reported across these studies is 32% [90% CI; 20.9–44.5%], as opposed to 3.7–9.4% by conventional karyotyping. This suggests that GS will not only increase the yield significantly, as shown by our study, more accurate molecular diagnoses can also be provided compared to conventional karyotyping.

Unlike single point mutations, there can be complex variations in BCAs and precise breakpoint detection can lead to more personalized genetic counseling and provide accurate information on prognosis and possible complications. Many of these genetic diagnoses will only lead to anomalies later in life and prenatal development may be completely normal. As shown by our case of possible infantile epilepsy and intellectual disability, accurate breakpoint information can be rather informative in the process of genetic counseling for the parents. As the cost of GS starts to reduce in the coming years, when faced with the decision of potential termination of pregnancy, it may be expected for clinical laboratories to provide this additional information. Therefore, GS should be considered early when designing the best prenatal diagnostic workflow for BCAs.

### Limitations

The study design only focused on cases with abnormal conventional karyotype results with sufficient DNA for GS. Although the sample size of this study is small, it is comparable with other literature published related to prenatal diagnosis and short read GS. Due to the retrospective nature of the study and complexity of Sanger validation in chromosomal breakpoint cases, only eight cases with enough DNA subjected to validation by orthogonal wet lab methods including Sanger sequencing, PacBio sequencing, and CMA. Yet for these eight cases with orthogonal confirmation, there was 100% concordance with the Illumina GS results.

Additionally, standard guidelines and tools for TAD analysis are not available, and in different TAD prediction tools, there are variable assumptions about TADs, such as the size distribution, type of Hi-C signal detected, and the presence or absence of overlap/nesting. The different assumptions made restrict the tools from predicting the full TAD landscape in cells therefore resulting in fairly discordant predictions for the TAD analysis (Dali and Blanchette, 2017). In this project, the tools AnnotSV and 3D Genome Browser were used for TAD analysis and the TAD annotated coordinates were listed in Supplementary Table 3. There were no findings by TAD analysis after looking into functional elements like enhancers and regulatory elements. More studies will be required on TAD analysis before it could be applied in prenatal diagnosis.

## Conclusion

To conclude, the findings from this study demonstrated the advantages of GS over conventional karyotyping on the detection of BCAs. GS allows the precise detection of BCA breakpoints and cryptic genomic imbalances surrounding the regions of BCAs. This results in better evaluation on the risk of congenital anomalies in BCA on a case-by-case basis. Compared to the previously stated 3.7–9.4% risk of congenital anomaly, our study demonstrated a yield of 40% with readily available short-read GS. Although the yield may be further increased with long read sequencing, we show that even by using short read GS pipelines, significant clinical utility can be gained by applying GS in clinical centers which may yet have the expertise to perform long-read sequencing.

## Data Availability Statement

The datasets for this article are not publicly available due to concerns regarding participant/patient anonymity. Requests to access the datasets should be directed to the corresponding author.

## Ethics Statement

The studies involving human participants were reviewed and approved by Institutional Review Board of the University of Hong Kong/Hospital Authority Hong Kong West Cluster (UW 18-045). Written informed consent to participate in this study was provided by the participants' legal guardian/next of kin.

## Author Contributions

MY was responsible for manuscript writing and data analysis as well as project coordination. JC helped with manuscript writing and data analysis. SA helped with the lab work. HL helped with the lab work. KY and CM helped with the data analysis. JF contributed the genetic counseling and clinical details. CC worked for a meta-analysis study. KC helped with the lab work and clinical cases. BC and AK supported the case recruitment, provided the clinical details, supervision, and financial support. All authors contributed to the article and approved the submitted version.

## Conflict of Interest

The authors declare that the research was conducted in the absence of any commercial or financial relationships that could be construed as a potential conflict of interest.
