# Supplementary material for: Evaluating the Clinical Utility of Genome Sequencing for Cytogenetically Balanced Chromosomal Abnormalities in Prenatal Diagnosis
Source: Front Genet. 2021 Jan 27;11:620162. doi: 10.3389/fgene.2020.620162 (PMC7873444; doi:10.3389/fgene.2020.620162)
Supplement: Supplementary file 1 [file Data_Sheet_1.DOCX]

**Supplementary methods**

**PacBio sequencing**

1X AMPure XP beads were used to purify the gDNA. 10 µg purified gDNA was processed for DNA shearing with Megaruptor 2. According to PacBio Preparing >30 kb SMRTbell Libraries workflow, 8 µg sheared DNA was used to generate SMRTbell library, and then the size selected DNA size >8kb by using BluePippin. The size selected SMRTbell library was sequenced on the PacBio Sequel system for 9 SMRTcells according to the manufacture’s instruction. The raw sequencing data were aligned to the human reference genome (hg19) by using Minimap2 (v2.12), and the structural variants were called by using Sniffles (v1.0.9).

**Karyotyping**

3-5 mg of dissected chorionic villi or 30mL amniotic fluid was obtained from invasive procedure for Giemsa banded (G-banded) karyotyping performed in Prenatal Diagnostic Laboratory, Tsan Yuk Hospital. Images of well spread and properly banded metaphases were captured using CytoVision (Leica Biosystem) automatic scanning system and visually examined by two independent experienced cytogeneticists.

**Search strategy for systematic review**

PubMed was used with the search terms "Prenatal" AND "balanced chromosomal" OR “BCA” AND "de novo" to identify relevant studies pertaining to short-read GS. Studies identified from citation matching were included in the systematic review. There were no language or date restrictions. Only results from cohort-based studies on short-read GS applied on prenatal samples were included in the analysis. Screening of the titles, abstracts, full text, and data extraction was performed by two authors independently. Discrepancies were identified, discussed, and resolved. Statistical analysis was performed using the R statistic software, version 3.5.0.​

**Table S1: Evaluation of GS breakpoint detection through orthogonal methods**

| **Case** |  | **Orthogonal method** | **Genomic breakpoints detected by GS**  **(GRCh37)** | **Derivative chromosome** | **Genomic breakpoints detected by orthogonal validation**  **(GRCh37)** | **SV Precisely detected by GS^#^** | **Insertion/deletion at breakpoint** |  |  |  |
| --- | --- | --- | --- | --- | --- | --- | --- | --- | --- | --- |
| 1 |  | Sanger | chr18:29,652,147  chr19:36,930,887 | der(18) | chr18:29,652,147  chr19:36,930,887 | ✓  ✓ | Nil |  |  |  |
|  |  |  |  | der(19) | chr18:29,652,158  chr19:36,930,879 | ✓  ✓ | Insertion of 19 bp |  |  |  |
| 2 |  | Sanger | chr4:186,776,072  chr12:15,513,829 | der(4) | Not tested | N/A | N/A |  |  |  |
|  |  |  |  | der(12) | chr4:186,776,072  chr12:15,513,829 | ✓  ✓ | Insertion of 11 bp |  |  |  |
| 5 |  | Sanger | chr1:100,004,147  chr4:88,309,179 | der(1) | chr1:100,004,147  chr4:88,309,179 | ✓  ✓ | Nil |  |  |  |
|  |  |  |  | der(4) | chr1:100,004,147  chr4:88,309,173 | ✓  ✓ | Deletion-insertion  (TCATTG>CAAAACTCTCTTT) |  |  |  |
| 6 |  | Sanger | chr8:97,086,794  chr11:65,540,889 | der(8) | Not tested | N/A | N/A |  |  |  |
|  |  |  |  | der(11) | chr8:97,086,794  chr11:65,540,885 | ✓  ✓ | Insertion of 1 bp |  |  |  |
| 8 |  | PacBio | chr21:14,953,345  chr21:47,839,992 | der(21) | chr21:14,953,346  chr21:47,839,992 | ✓ | Nil |  |  |  |
| 9 |  | Sanger | chr6:45,841,060  chr8:126,545,471 | der(6) | chr6:45,841,061  chr8:126,545,471 | ✓  ✓ | Insertion of 2 bp |  |  |  |
|  |  |  |  | der(8) | chr6:45,841,060  chr8:126,545,471 | ✓  ✓ | Nil |  |  |  |

*#: “Precisely detected” is defined as breakpoint validation detected within 20bp of GS breakpoint detection.*

**: “Not tested”due to not enough DNA to do Sanger sequencing validation*

**Table S2: Primer sequences used in Sanger sequencing validation of breakpoints**

| Case | derivative  chromosome. | Forward Primer | Reverse Primer |
| --- | --- | --- | --- |
| 1 | der(18) | der18-F1: TGCACACCTTTAGTTTCAGCT | der18-R1:  ATGAGCCAGCCACCACAC |
|  | der(19) | der19-F1: AGAGCACCTTCACCAGAGAC | der19-R1:  GTGCCTTTTGTATCGCTCTGA |
| 2 | der(12) | der12 F1:  AAAACTATGCCCTGGACCAG | der12 R1:  GCCCGACCCATGTTCTGAAT |
| 5 | der(1) | der1-F1: TGTGAGTGGAAATACATGCTGG | der1-R1:  TGCATGCCAAGAAGATGACG |
|  | der(4) | der4-F1: CCCGGTTCAATAGTCCCCAT | der4-R1:  TCAGTCCTCACAGTTGGCTT |
| 6 | der(11) | der11-F1: AGGAGGGGTTAGGGTAGGAG | der11-R1:  GCTGAGACGATGGGGTTTTC |
| 9 | der(6) | der6-F1: AGGCAGTCTCTAAAGCGGTC | der6-R1:  TGGCGAAGAAAGGTGTTTGA |
|  | der(8) | der8-F1: TGTGACTTGCTCCTCCTTGT | der8-R1:  AGCCCCTTCAGCCTTGTTAT |

Breakpoint PCR primers were designed by retrieving 1kb genomic sequences flanking the putative breakpoint from the UCSC genome (GRCh37/hg19). Primers were designed using Primer 3 tool and blasted on NCBI for specificity. For each derivative chromosome, 1 pair of primers were used for PCR and Sanger sequencing. In brief, 100 ng genomic DNA was used as a template and PCR was performed using the FastStart™ High Fidelity PCR System (Roche) according to the manufacturer’s protocol. Amplicons were sequenced using the BigDye™ Terminator v3.1 Cycle Sequencing Kit (Applied Biosystems) and analyzed on the 3500xL Genetic Analyzer (Applied Biosystems).

**Table S3: Detailed clinical information, BCA breakpoint, CNV deletions, and mutation classification of all ten cases of this study**

**See attachment of Table S3.xlsx**

**Figure S1 – Analysis workflow of this study**


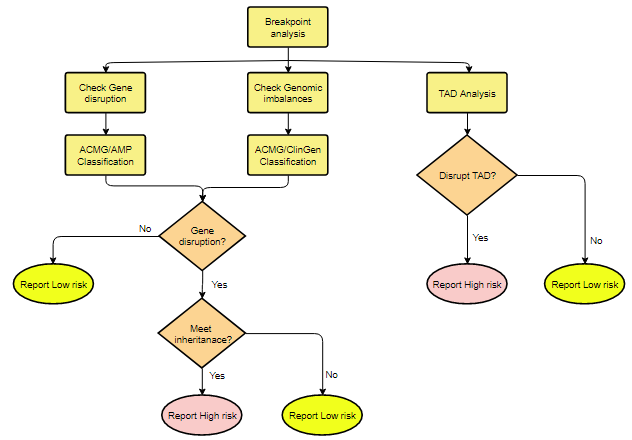


The above diagram showed the breakpoint analysis workflow of this study. All ten cases with BCA were analyzed on three aspects – gene disruption, genomic imbalances, and TAD analysis. The ACMG/AMP and ACMG/ClinGen guidelines were adopted for the interpretation and reporting of the sequence variants and Copy-Number Variants. The outcome was to report “High or Low” risks after relating to the gene-disease association and genotype-phenotype correlation.

Abbreviation:

TAD = topologically associated domain

Rectangle block – Analyzing data

Diamond block – Making a decision

Eclipse block – Reporting outcome

Red Eclipse block –Reporting High risk

Amber Eclipse block – Reporting Low risk

**Figure S2 –Analysis workflow of Topologically associated Domains (TAD)**


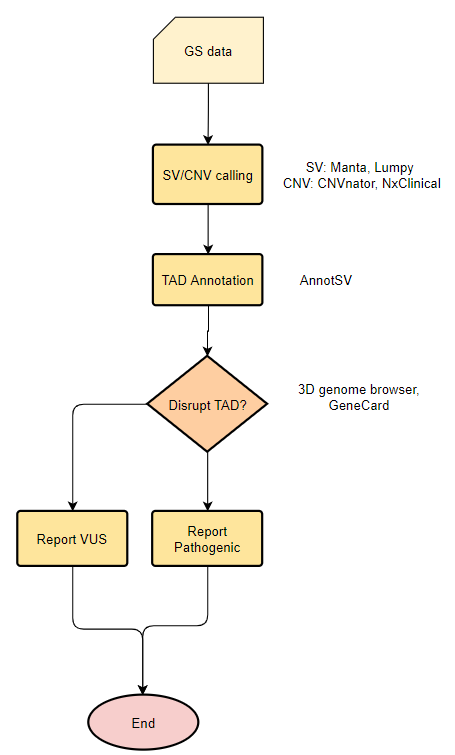


The above diagram showed the analysis of TAD in this study. Previous studies have shown that SV and CNV affecting boundaries of TADs could alter the architecture and enhancer-promoter interactions within TADs thereby causing diseases. (Ibn-Salem et al., 2014;dos Santos et al., 2020)

In this study, we analyzed the TAD regions annotated by AnnotSV based on the SV and CNV detected by the bioinformatic tools (Manta, Lumpy, CNVnator and NxClinical). The captured Hi-C data from various human cell lines was provided by the Penn State 3D genome browser. We manually examined whether any SV or CNV disrupted any enhancer or promoter regions of OMIM morbid genes. This was performed by comparing the genomic coordinates of the SV or CNV and the annotated TADs or enhancer/promoter on the web resources such as GeneCards. Finally, we double checked with evidence and curation on haploinsufficiency and triplosensitivity performed by ClinGEN and other reported public resources showing deletion or duplication is a disease-causing mechanism.

URL of the tools:

3D genome browser: <http://3dgenome.fsm.northwestern.edu/>

AnnotSV: <https://www.lbgi.fr/AnnotSV/>

GeneCards: <https://www.genecards.org/>

ClinGEN: <https://clinicalgenome.org/>

**Figure S3: Sanger sequencing validation of derivative chromosomes in five cases**

1. **Case 1 with karyotype 46,XX,t(18;19)(q12.2;q13.1)dn**


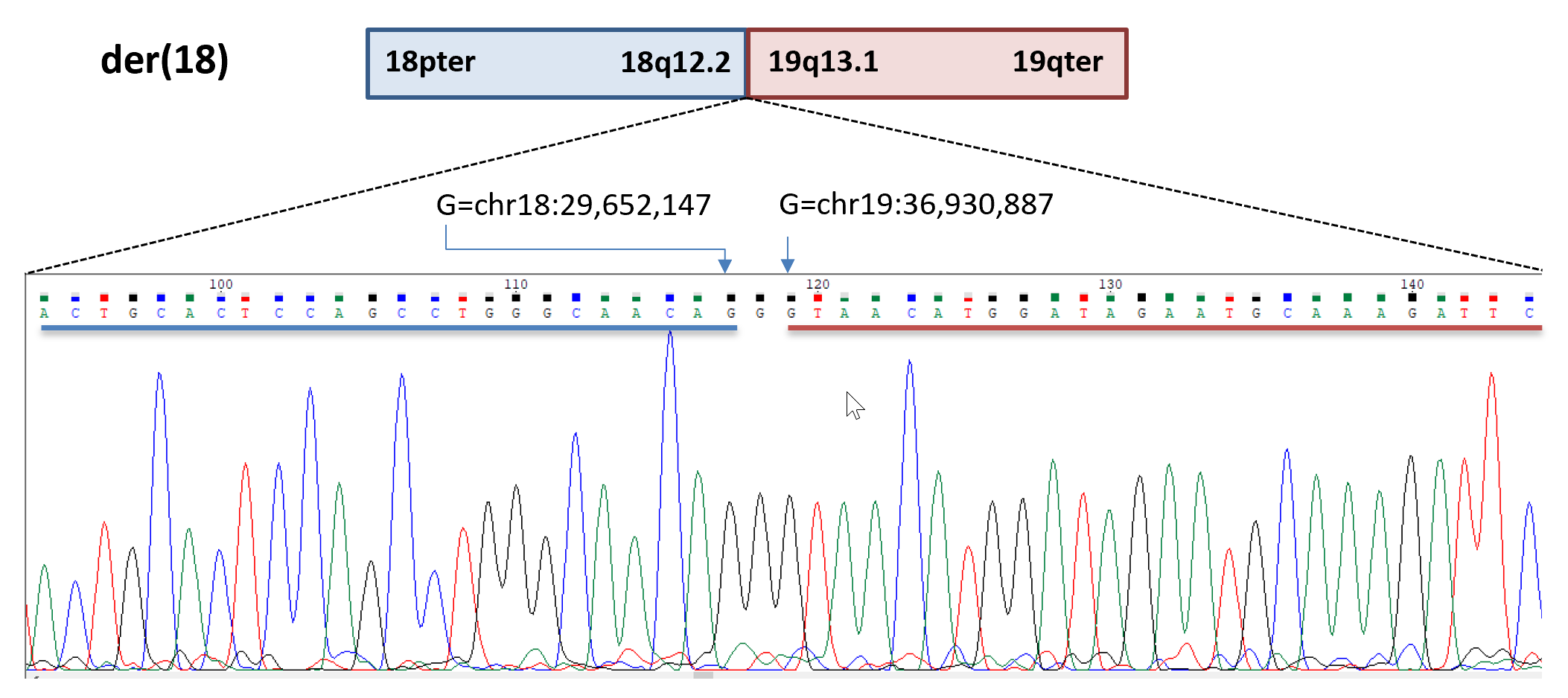


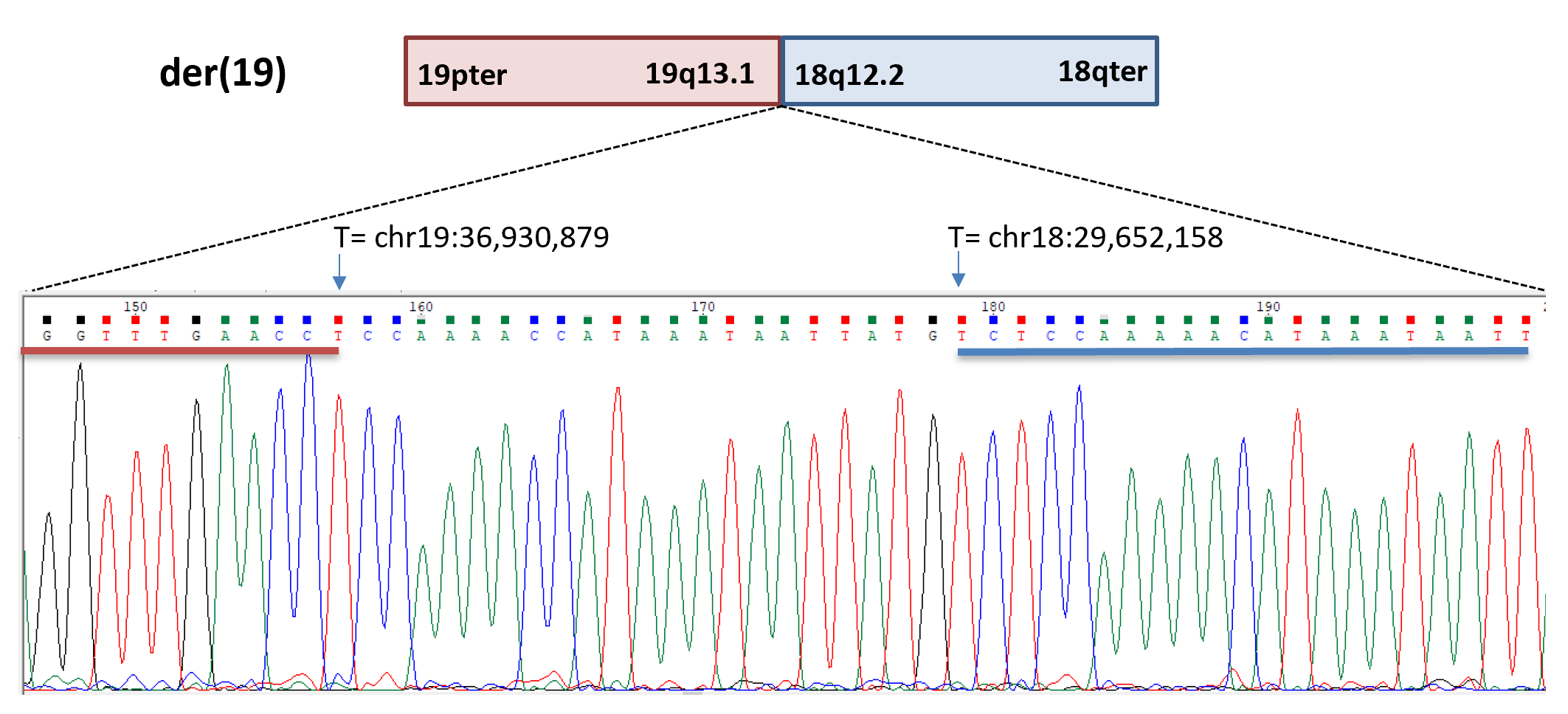


**(B) Case 2 with karyotype 46,XX,t(4;12)(q35;p13.1)dn**


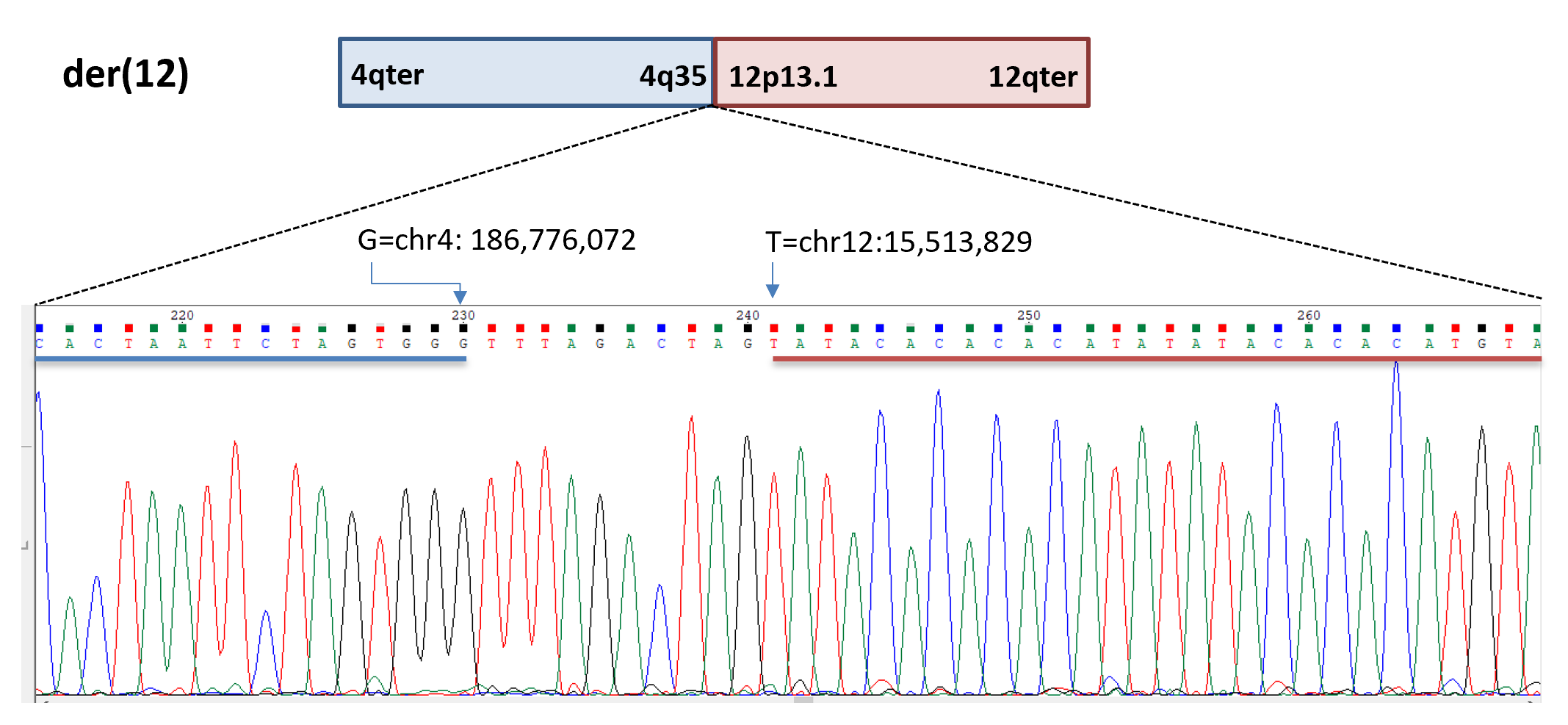


**(C) Case 5 with karyotype 46,XY,t(1;4)(p21;q21.1)dn**


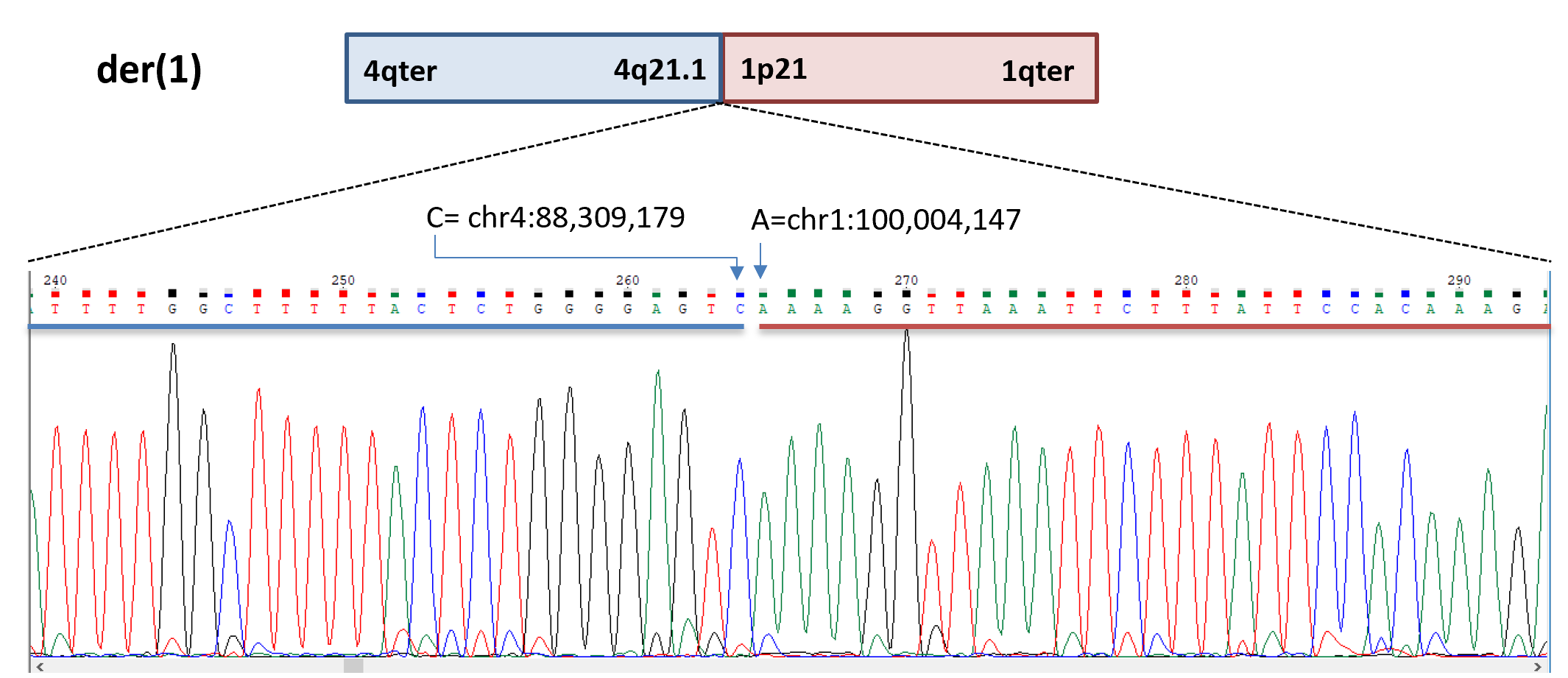


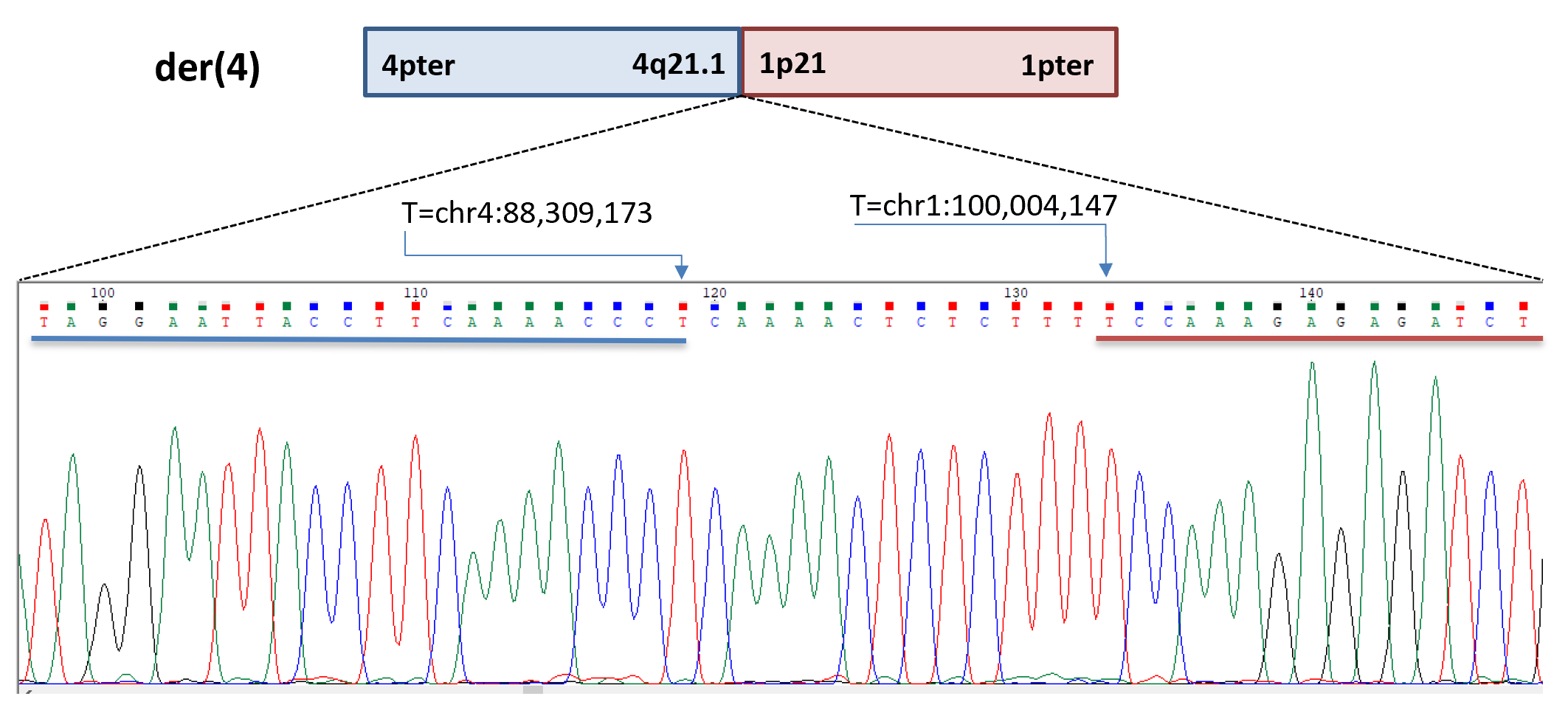


**(D) Case 6 with karyotype 46,XX,t(8;11)(q22;q13)dn**


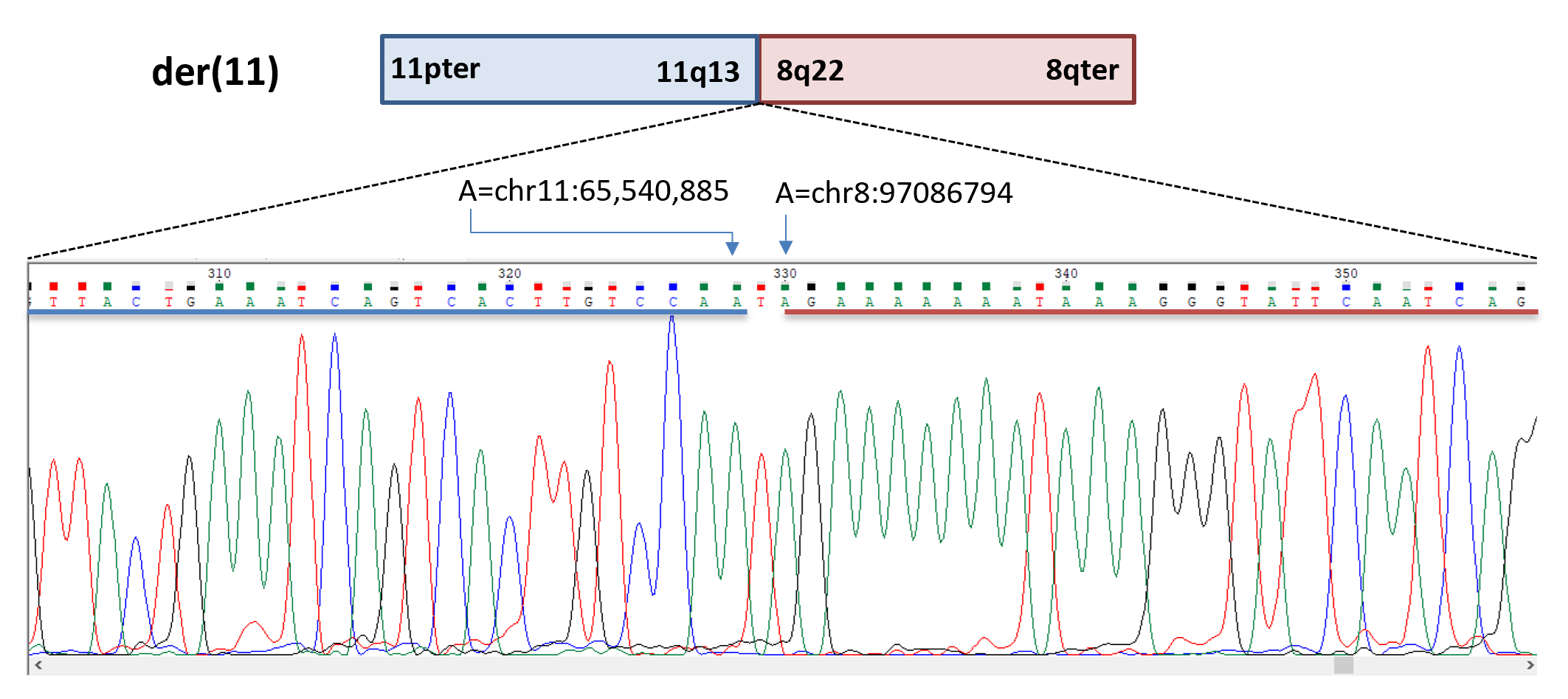


**(E) Case 9 with karyotype 46,XY,t(6;8)(p21.1;q24.1)dn**


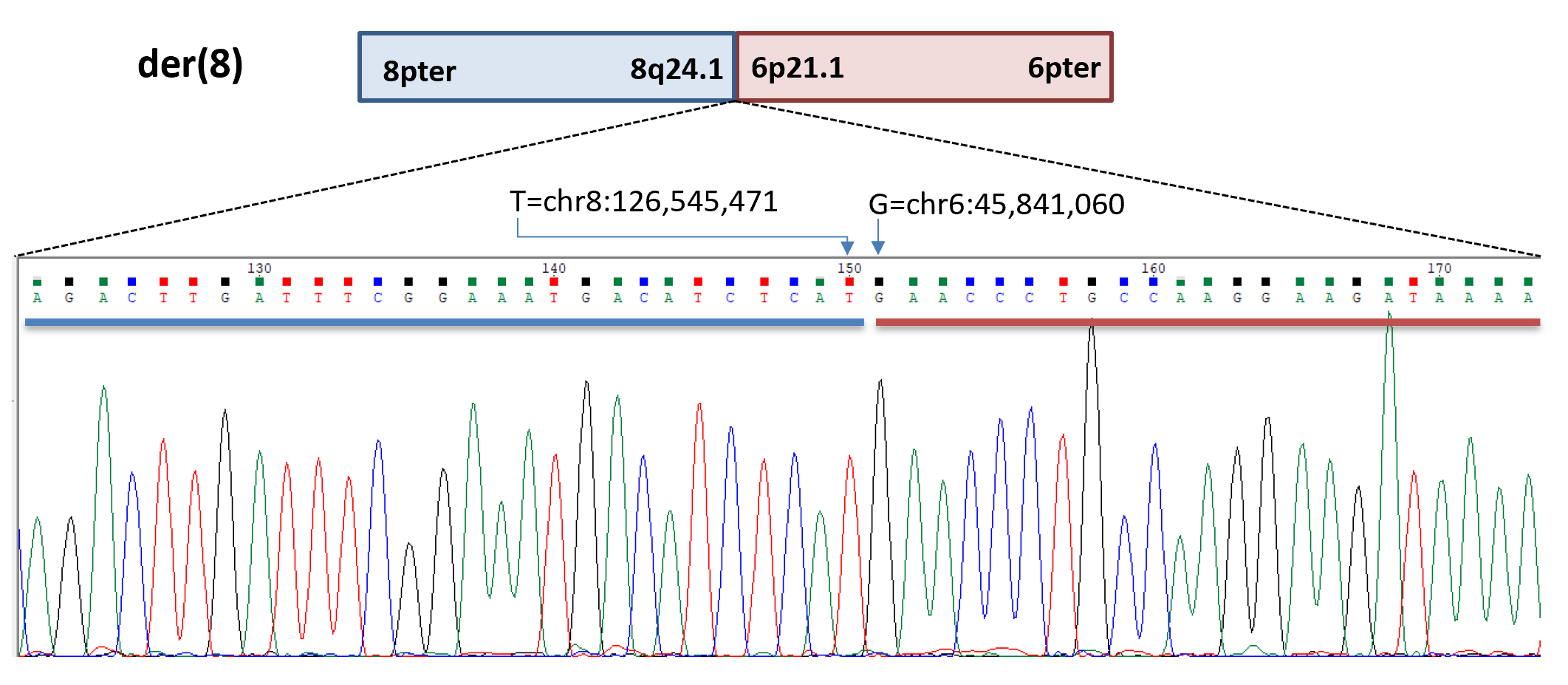


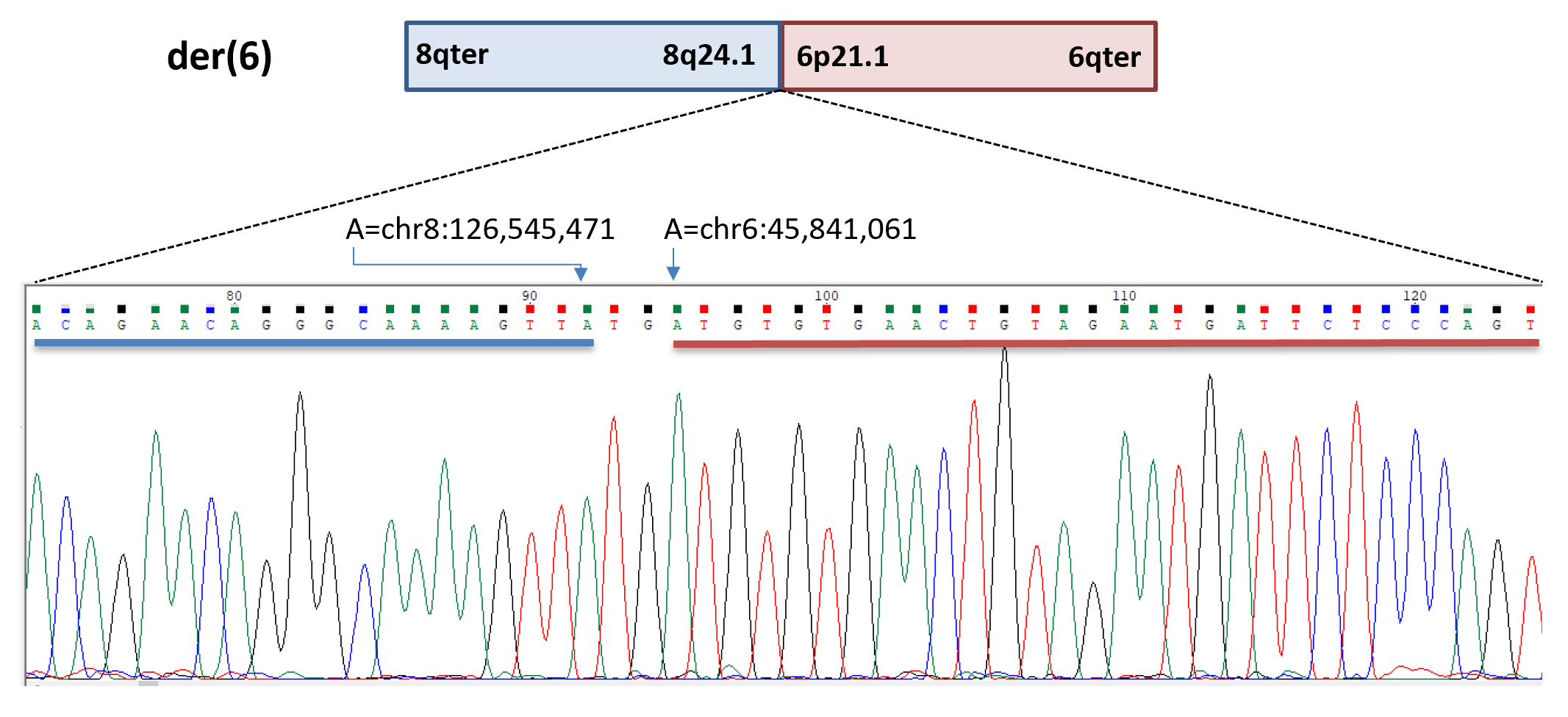


Sanger sequencing validation for the analytical validity of genome sequencing in determining the balanced chromosomal abnormality breakpoints in Case 1 (A), Case 2 (B), Case 5 (C), Case 6 (D) and Case 9 (E). Note that der(4) in (B) and der(8) in (D) are not available due to insufficient DNA to perform Sanger sequencing validation. Sanger sequencing traces with arrows indicating the nucleotide position of the breakpoints determined by WGS. The nucleotides between arrows indicated microinsertion near breakpoint, if there is any. Derivative (der) chromosomes showing the rearranged segments were represented as blue/red colored boxes (not in scale).

**Figure S4** - Workflow of detecting phasing by WhatsHap and short read and long read sequencing data


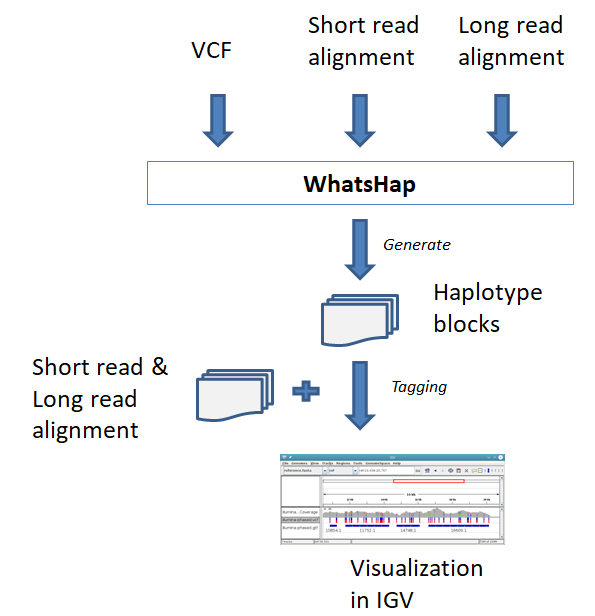


WhatsHap is a read-based phasing assembly tool. Long-read (PacBio) and short-read (Illumina) sequencing reads were used to reconstruct the haplotypes. A VCF file is outputted augmented with phasing information that haplotype block and haplotype information will be generated. A GTF file is also created from a phased VCF file that describes the haplotype blocks and is visualized in IGV.

**Figure S5:** **Pedigree of Case 8**

42y

37y

Heterozygous *PCNT*:c.4633_4678delAGACAAGTGTTAAT:p.(R1555Afs*6)

7y

Heterozygous *PCNT*:c.4633_4678delAGACAAGTGTTAAT:p.(R1555Afs*6), inherited from mother

Heterozygous seq[GRCh37] inv(21)(pter->q11.2::q22.3->q11.2::q22.3->qter)dn

chr21:g.[14953345_47839992inv]

Pedigree of Case 8 shows the inheritance pattern of the two mutations found in the child. The mother is heterozygous for a frameshift mutation in *PCNT*:c.4633_4678delAGACAAGTGTTAAT:p.(R1555Afs*6) which was passed on to the child. GS showed an apparent *de novo* BCA breakpoint in the *PCNT* gene causing *PCNT* gene disruption on the other allele

**Figure S6: IGV of the inversion breakpoint of Case 8 on sequence reads generated from Illumina and PacBio sequencer**

1. Inversion breakpoint at chr21:14,953,345


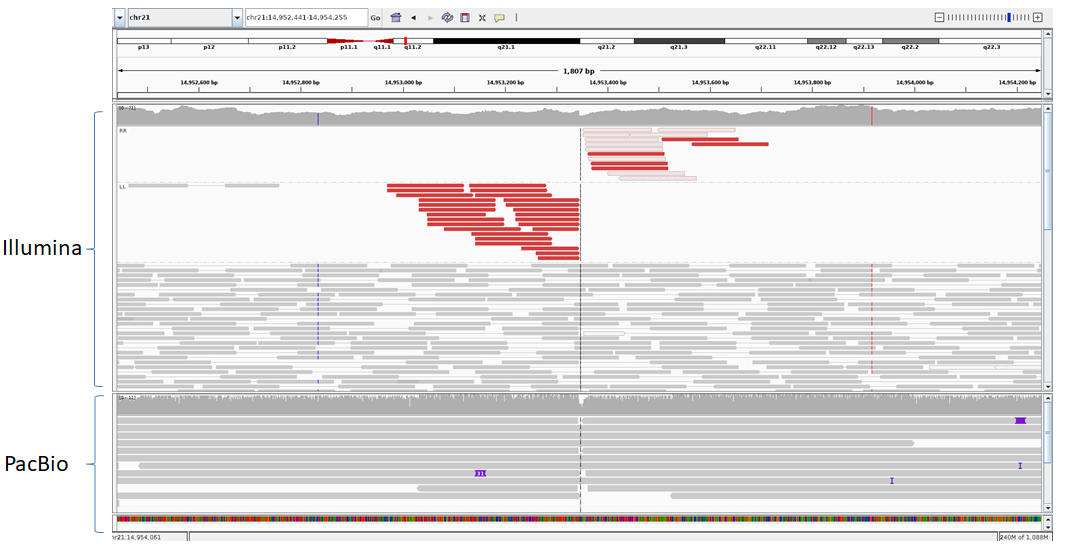


1. Inversion breakpoint at chr21:47,839,992


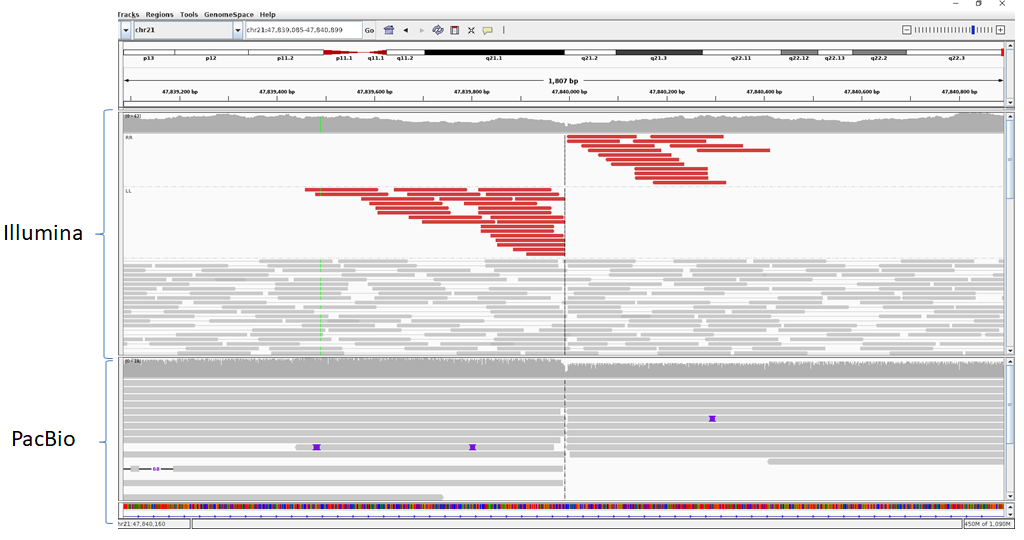


Both IGV plots show the BCA inversion breakpoints at chromosome 21 of case 8 and each has two tracks showing sequence reads of two sequencing platforms - Illumina (top) and PacBio (bottom). The sequence reads are formatted by “Group alignments by pair orientation" in IGV. All the coverage tracks of Illumina and PacBio track at the inversion breakpoints show the acute depth of coverage drop. The red color sequence reads shown in the upper track (Illumina) of both (A) and (B) illustrate missing partner reads beyond the breakpoint (vertical black line). Split reads were also shown in the PacBio tracks at the breakpoints. Breakpoints and the genomic coordinates matched with those predicted by bioinformatics tools.

**Figure S7 – Comparing this study with other published studies using GS to detect and characterize BCA in Prenatal setting.**


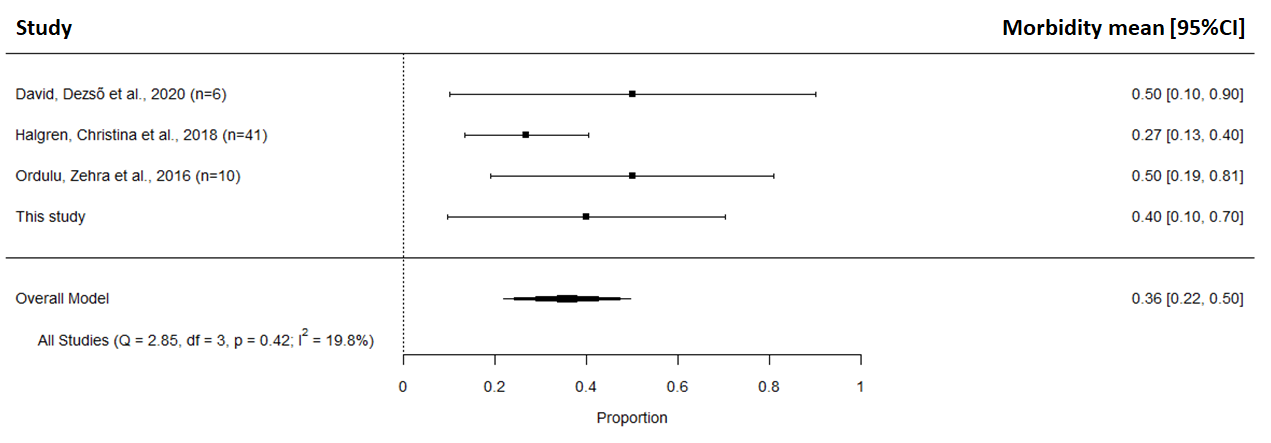


In four studies comprising 67 patients, the pooled morbidity was found to be 36% (95% CI 22%–50%, I^2^=19.8%) using a random-effects model, ranging from 27% (n=41) to 50% (n=10). The low I^2^ score showed low between-study heterogeneity.(Ordulu et al., 2016;Halgren et al., 2018;David et al., 2020)

**Supplementary references:**

David, D., Freixo, J.P., Fino, J., Carvalho, I., Marques, M., Cardoso, M., Piña-Aguilar, R.E., and Morton, C.C. (2020). Comprehensive clinically oriented workflow for nucleotide level resolution and interpretation in prenatal diagnosis of de novo apparently balanced chromosomal translocations in their genomic landscape. *Hum Genet* 139**,** 531-543.

Dos Santos, A.P., Meinel, J.A., Cruz Piveta, C.D.S., De Andrade, J.G.R., Fabbri-Scallet, H., Gil-Da-Silva-Lopes, V.L., Guerra-Júnior, G., Künstner, A., Kaiser, F.J., Holterhus, P.-M., Hiort, O., Busch, H., Maciel-Guerra, A.T., De Mello, M.P., and Werner, R. (2020). Disruption of the topological associated domain at Xp21.2 is related to gonadal dysgenesis: A general mechanism of pathogenesis. *medRxiv***,** 2020.2003.2025.20041418.

Halgren, C., Nielsen, N.M., Nazaryan-Petersen, L., Silahtaroglu, A., Collins, R.L., Lowther, C., Kjaergaard, S., Frisch, M., Kirchhoff, M., Brøndum-Nielsen, K., Lind-Thomsen, A., Mang, Y., El-Schich, Z., Boring, C.A., Mehrjouy, M.M., Jensen, P.K.A., Fagerberg, C., Krogh, L.N., Hansen, J., Bryndorf, T., Hansen, C., Talkowski, M.E., Bak, M., Tommerup, N., and Bache, I. (2018). Risks and Recommendations in Prenatally Detected De Novo Balanced Chromosomal Rearrangements from Assessment of Long-Term Outcomes. *Am J Hum Genet* 102**,** 1090-1103.

Ibn-Salem, J., Köhler, S., Love, M.I., Chung, H.R., Huang, N., Hurles, M.E., Haendel, M., Washington, N.L., Smedley, D., Mungall, C.J., Lewis, S.E., Ott, C.E., Bauer, S., Schofield, P.N., Mundlos, S., Spielmann, M., and Robinson, P.N. (2014). Deletions of chromosomal regulatory boundaries are associated with congenital disease. *Genome Biol* 15**,** 423.

Ordulu, Z., Kammin, T., Brand, H., Pillalamarri, V., Redin, C.E., Collins, R.L., Blumenthal, I., Hanscom, C., Pereira, S., Bradley, I., Crandall, B.F., Gerrol, P., Hayden, M.A., Hussain, N., Kanengisser-Pines, B., Kantarci, S., Levy, B., Macera, M.J., Quintero-Rivera, F., Spiegel, E., Stevens, B., Ulm, J.E., Warburton, D., Wilkins-Haug, L.E., Yachelevich, N., Gusella, J.F., Talkowski, M.E., and Morton, C.C. (2016). Structural Chromosomal Rearrangements Require Nucleotide-Level Resolution: Lessons from Next-Generation Sequencing in Prenatal Diagnosis. *Am J Hum Genet* 99**,** 1015-1033.
